# Translational Application of Fluorescent Molecular Probes for the Detection of Reactive Oxygen and Nitrogen Species Associated with Intestinal Reperfusion Injury

**DOI:** 10.3390/metabo11120802

**Published:** 2021-11-26

**Authors:** Gustavo Sampaio de Holanda, Samuel dos Santos Valença, Amabile Maran Carra, Renata Cristina Lopes Lichtenberger, Bianca de Castilho, Olavo Borges Franco, João Alfredo de Moraes, Alberto Schanaider

**Affiliations:** 1 Centre of Experimental Surgery, Post Graduate Program in Surgical Sciences, Department of Surgery, Faculty of Medicine, Federal University of Rio de Janeiro, Rio de Janeiro 219491-590, Brazil; amabilemcarra@gmail.com (A.M.C.); renata.2001berger@gmail.com (R.C.L.L.); biacaastilo@gmail.com (B.d.C.); olavobf1988@gmail.com (O.B.F.); albertoscha@gmail.com (A.S.); 2 Redox Biology Laboratory, Institute of Biomedical Sciences, Federal University of Rio de Janeiro, Rio de Janeiro 21941-901, Brazil; samuelv@icb.ufrj.br (S.d.S.V.); joaomoraes@icb.ufrj.br (J.A.d.M.)

**Keywords:** ischemia-reperfusion injury, molecular probes, oxidative stress, mesenteric ischemia

## Abstract

Acute mesenteric ischemia, caused by an abrupt interruption of blood flow in the mesenteric vessels, is associated with high mortality. When treated with surgical interventions or drugs to re-open the vascular lumen, the reperfusion process itself can inflict damage to the intestinal wall. Ischemia and reperfusion injury comprise complex mechanisms involving disarrangement of the splanchnic microcirculatory flow and impairment of the mitochondrial respiratory chain due to initial hypoxemia and subsequent oxidative stress during the reperfusion phase. This pathophysiologic process results in the production of large amounts of reactive oxygen (ROS) and nitrogen (RNS) species, which damage deoxyribonucleic acid, protein, lipids, and carbohydrates by autophagy, mitoptosis, necrosis, necroptosis, and apoptosis. Fluorescence-based systems using molecular probes have emerged as highly effective tools to monitor the concentrations and locations of these often short-lived ROS and RNS. The timely and accurate detection of both ROS and RNS by such an approach would help to identify early injury events associated with ischemia and reperfusion and increase overall clinical diagnostic sensitivity. This abstract describes the pathophysiology of intestinal ischemia and reperfusion and the early biological laboratory diagnosis using fluorescent molecular probes anticipating clinical decisions in the face of an extremely morbid disease.

## 1. Introduction

Acute mesenteric ischemia (AMI) is characterized by an abrupt interruption or reduction of the intestinal blood supply, either temporarily or permanently, and is a medical-surgical emergency that requires immediate attention. Despite advances in medical imaging techniques, the evolution of clinical knowledge, and the development of biotechnology beyond the adoption of less invasive treatments, AMI still represents a major diagnostic and therapeutic challenge, largely due to the absence of specific markers related to the severity of the condition [1,2,3]. The clinical course of the disease is correlated with a high mortality rate (70%) in patients with AMI admitted to the emergency room [4].

It should be clarified that in clinical practice mesenteric ischemia is more severe when the occlusion occurs more centrally in a mesenteric vessel. Mechanical obstruction is the most common etiology of ischemia, followed by intestinal reperfusion. It can be caused by an embolus or thrombus within the vascular lumen, but also by phenomena that directly affect the intestinal wall such as volvulus (loop twist), intestinal invagination, incarcerated hernia, or extrinsic compression (caused by tumors or adhesions among others). In addition to a mechanical obstruction, an intestinal transplant, blood pressure variations (hypovolemic, cardiogenic, or neurogenic shock), and non-occlusive phenomena (e.g., due to the use of drugs) represent other reported causal events [3].

The quicker appropriate treatment of AMI is provided, the greater the chances of successful visceral perfusion with the return of effective blood flow and reduced risk of any sequelae. However, reperfusion inevitably produces reactive oxygen (ROS) and nitrogen (RNS) species, indicative of oxidative and nitrosative stress, respectively. The severity of intestinal tissue damage due to increased ROS and RNS levels depends on the magnitude of exacerbated synthesis of these humoral mediators, which is determined by the persistence of the deleterious causal agent and the extent of depletion of the organic defensive capacity to scavenge such reactive species. Under conditions of sustained stress, degradation of energy sources, enzyme synthesis, and activation of nuclear transcriptional factors occur, resulting in a chain reaction with significant production of ROS and/or RNS and the formation of several oxidizing substances, including lipid peroxides and carbonyl proteins. Such oxidants can trigger extensive cell damage and aggravate ischemia-initiated injury in the intestinal loop [5]. Beyond their roles in processes like modulation of cell survival, differentiation, cell death, cell signaling, and inflammation-related factor production, some reactive species have clear beneficial actions, such as the containment of invading pathogens, when present in physiologically ‘normal’ levels. However, when overproduced they typically become harmful to cellular homeostasis and can cause local and distant tissue damage [6].

Currently, the diagnosis of mesenteric ischemia is based on patient history, main symptoms, physical examination findings, and the use of state-of-the-art imaging methods. Abdominal computed tomography angiography with three-dimensional imaging can identify features of acute splanchnic vascular obstruction and intestinal injury. However, it is inaccurate in assessing the extent and severity of parietal involvement [7]. In this context, there is a lack of serum biomarkers and/or molecular methods to identify AMI with satisfactorily specificity and sensitivity to enable a straightforward and rapid diagnosis when required (i.e., as soon as AMI is suspected) [8].

Fluorescent probes allow for accurate detection of complex biomolecular components, such as ROS and RNS. Taking advantage of the diagnostic potential of these probes in diseases that affect visceral perfusion can benefit clinical-surgical practices, especially when it comes to patients with early acute abdominal pain. The present review will explore fluorescence techniques that rely on molecular probes for the measurement of ROS and RNS and evaluate how they could serve as a useful complementary resource in the diagnosis of AMI. 

## 2. Discussion

### 2.1. Historical Context

The first studies related to oxygen-derived free radicals date back to 1931 when Haber and Weiss described the monovalent reduction of molecular oxygen to superoxide anion (O_2_^−^), a free radical capable of oxidizing organic structures and enzymes [9]. The conversion of superoxide to the highly reactive hydroxyl radical (OH**^.^**) was described three years later [10].

In 1968, McCord and Fridovich referred to xanthine oxidase (XO) as a biological source of superoxide production and found it was present in various organic tissues. The same authors later described the discovery of the superoxide dismutase enzyme capable of inactivating the superoxide radical [11]. In the 1970s, N-acetylcysteine, a small molecule inclosing the thiol group, was identified as a ROS scavenger, acting as a potent antioxidant [12].

It was not until the early 1980s that the inexplicable worsening of patients after supposedly adequate treatment to relieve AMI was starting to be clarified in terms of pathophysiology, biochemistry, and molecular biology. The first reports on potentially harmful effects of intestinal reperfusion were related to cell damage. De novo oxygenation of ischemic tissues was found to produce ROS and cause deleterious effects on cell structures by lipid peroxidation, protein oxidation, and nucleotide involvement, including purine bases [13,14,15,16]. In 1981, Granger and colleagues characterized the presence of XO in the intestine of different animals and humans and determined its relationship with tissue damage following intestinal ischemia and reperfusion. These data were fundamental in sparking research focusing on the role of reactive species biology in the digestive system under physiological and pathological conditions [17,18,19].

During the 1980s, endovascular intervention using catheters in the vessel lumen to locally deliver drugs therapy or for revascularization of AMI emerged as an alternative approach. It is a less invasive procedure than surgically opening the abdominal cavity and, if successful, reduces the need for intestinal resection, incidence of postoperative complications, length of hospitalization and mortality [20,21].

In the mid-1980s, the first probes consisting of a single-stranded deoxyribonucleic acid (DNA) fragment conjugated to a product (radioisotope, biotin, fluorescent compound) were developed. Nevertheless, fluorescent molecular probes to study oxidative stress and detect specific intracellular components in complex biomolecular matrices, with applicability in various visceral ischemic conditions, were only introduced very recently [22].

### 2.2. Free Radical Synthesis and the Pathophysiology of Ischemia/Reperfusion

**Figure 1:**
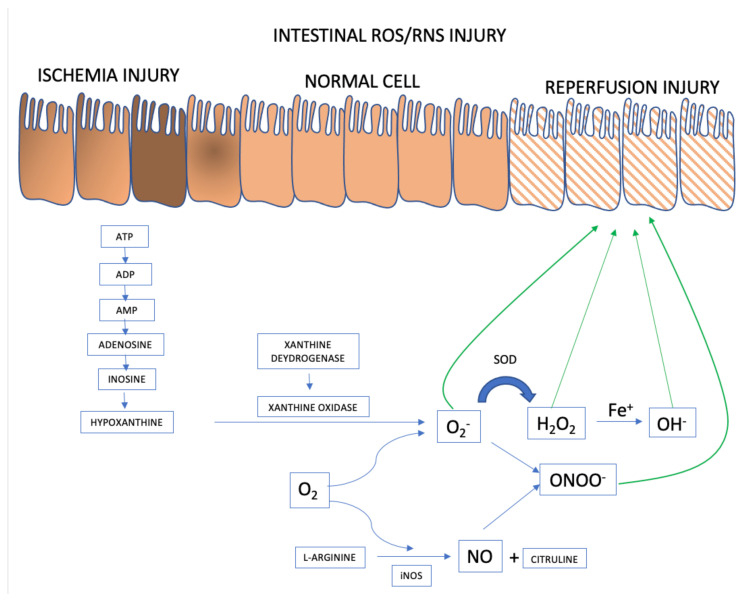
ROS and RNS formation mechanisms in the AMI setting. Adapted from [23].

When (partial or total) occlusion of the superior mesenteric artery or its branches occurs, splanchnic perfusion is limited due to reduction or, more frequently, interruption of blood flow. Blockage of oxygen supply and an impediment to aerobic energy metabolism induce an acute pathophysiological changes in the affected tissue(s) [23]. The lack of oxygen supply causes tissue ischemia and, if not restored promptly, will result in cellular dysfunction and cell death, ultimately resulting in parietal necrosis [24,25] (Figure 1).

#### Intestinal Epithelial Cells

ROS and RNS formation begins in the intestinal ischemia phase with adenosine triphosphate (ATP) accumulation generated in the anaerobic metabolism. There is degradation until the accumulation of hypoxanthine that at the beginning of the reperfusion phase, when there is reintroduction of oxygen to the intestinal tissue, interact with xanthine oxidase forming the superoxide anion (O_2_^−^), the first ROS formed. From there, the organism launches defenses such as superoxide dismutase (SOD), attenuating and forming ROS such as hydrogen peroxide (H_2_O_2_). However, if the response to reperfusion injury continues, the hydrogen peroxide is transformed into hydroxyl from the metal Iron (Fe^+^) into hydroxyl (OH^−^), in the so-called Fenton reaction. And in parallel, there may be the activation of RNS with the formation of nitric oxide (NO) from L-arginine, mediated by inducible nitric oxide synthase (iNOS). The combination of NO and superoxide anion forms the highly reactive species called nitrite peroxide (ONOO) which will further damage the intestinal cell’s epithelium.

During the ischemic phase, mitochondrial oxidative phosphorylation is inhibited rendering a drop in the production and storage of adenosine triphosphate (ATP). ATP is successively degraded to adenosine diphosphate (ADP), adenosine monophosphate (AMP), adenosine, inosine, and finally hypoxanthine. Lack of cellular energy causes sodium-potassium (Na^+^/K^+^) pump failure resulting in intracellular Na^+^ accumulation and K^+^ out of cells, ultimately leading to cellular edema and organelle dysfunction. In addition, an influx of calcium (Ca^2+^) and chloride (Cl^−^) ions into the intracellular environment occurs and triggers the activation of calpain protease, which in turn promotes the breakdown of a peptide bridge of the enzyme xanthine dehydrogenase (XDH) and subsequent formation of XO.

Although essential for the rescue of morphofunctional integrity of the affected tissues, restoration of mesenteric blood flow and consequent ischemic tissue reoxygenation has a deleterious effect because, paradoxically, reperfusion itself aggravates the damage [26,27]. Oxygen together with hypoxanthine and XO, synthesized during ischemia, catalyze the formation of ROS [28,29]. Re-introducing oxygen into the visceral circulation via reperfusion leads to the formation of O_2_^−^ and hydrogen peroxide (H_2_O_2_) after successive monovalent reductions. In the presence of iron, copper, cobalt, chromium, or vanadium, the production of highly reactive hydroxyl radical (OH**^.^**) is promoted via the Haber-Weiss and Fenton reactions [30]. There is an activity burst of the oxidative process characterized by the abundant production of multiple ROS and RNS within a few minutes after the restoration of blood flow [27]. The events underlying the damage caused by ischemia/reperfusion produce an uncontrolled and excessive release of ROS and RNS that overcome the organic line of defense represented by free radical scavengers [31]. 

The mitochondrial respiratory electron transport chain is the main intracellular site of ROS production and polymorphonuclear leukocytes play an important role in several pathological conditions also generating free radicals and nitric oxide (NO) synthesis. Different forms of mitochondrial dysfunction and tissue inflammation can affect the organ undergoing ischemia and reperfusion and may even compromise other organs and systems with a paracrine or and endocrine effect. This phase can lead to the failure of multiple organs and systems [23,32]. 

Nitric oxide (NO) dynamics underpin changes involving RNS. NO is produced from L-arginine by three main isoforms of nitric oxide synthase (NOS): epithelial NOS (eNOS), related to vasodilation and vascular regulation; neuronal NOS (nNOS), linked to various intracellular signaling pathways; and inducible NOS (iNOS), which has been reported to have beneficial microbicidal, antiviral, antiparasitic and antitumoral actions, but has also been implicated in the pathophysiology of colitis [33]. While the production of NO by nNOS and eNOS is regulated by a Ca^2+^/calmodulin-dependent mechanism, iNOS is activated in response to triggers such as endotoxins or cytokines, which can lead to rapid production of large amounts of NO. Several diseases have been associated with excessive levels of NO production, resulting in serious deleterious cell-physiological consequences [34,35,36,37,38]. All products formed by NO reactions are collectively called RNS. Despite the discovery of NO as an endothelium-derived relaxing factor, it plays a critical role in the pathophysiology of sepsis as an important mediator of endotoxin-induced arteriolar vasodilatation, hypotension, and shock [39]. At high concentrations, NO is importantly involved in inflammatory, infectious, and degenerative diseases [40]. Via reactions with other free radicals produced during oxidative stress, NO can be converted to nitrogen dioxide (NO_2_), peroxynitrite (ONOO^−^), and dinitrogen trioxide (N_2_O_3_). NO_2_ is formed from NO autoxidation (reaction of NO with oxygen). ONOO^−^ is a powerful electron oxidant and is formed through the diffusion-controlled reaction between O_2_^−^ and NO; its most relevant targets are peroxiredoxins, glutathione peroxidase (GSH), CO_2_, and metal centers. N_2_O_3_ can be formed from a reaction between NO_2_ and NO and is considered an important intermediate in the autoxidation of NO. N_2_O_3_ is rapidly hydrolyzed to NO_2_ [41]. All these compounds can subsequently react with various classes of biomolecules, including lipids, DNA, thiols, amino acids, and metals, leading to oxidation and nitration. If produced at high levels, RNS will detrimentally impact cell function, leading to changes in membrane integrity, loss of enzyme function, and DNA mutations [42]. 

It is noteworthy that, despite its typically beneficial antioxidant and vasodilatory functions, NO in high concentrations induces caspase-mediated apoptosis of epithelial cells in the intestinal tissue during ischemia and reperfusion. In addition, O_2_^−^ rapidly reacts with NO to produce ONOO^−^, which is another potent oxidant [43]. In the vasculature, the reaction of NO with O_2_^−^ leads to the formation of ONOO^−^ and decreases the vasorelaxant efficacy of NO. ONOO^−^ is a strong oxidant that can hydroxylate aromatic amino acids, oxidize thiols and lipids, and nitrate-free and protein-bound tyrosine residues. The number of possible reactions leading to secondary RNS formation illustrates the strong potential of NO to contribute to oxidative damage. High concentrations of NO, particularly in combination with increased oxidant production, cause tissue damage and inflammation through the production of NO_2_, ONOO^−^ and other nitrating, nitrosating, and oxidizing intermediates, and via inhibition of metal-dependent enzymes [44,45].

Several enzymes, such as cytochrome P450, the enzyme complexes of the mitochondrial respiratory chain, XO [46], eNOS [47], heme oxygenase (HO) [48], myeloperoxidase (MPO) [49], lipoxygenase (LOX), cyclooxygenase (COX) [50], and NADPH oxidases (NOX) [51] generate ROS under pathological conditions leading to oxidative stress [52]. All these factors contribute to persistent oxidative stress in the cellular environment, which will result in progressive functional impairment of critical intracellular organelles and structures, including membranes, mitochondria, the endoplasmic reticulum, the cytoskeleton, and the nucleus. These deleterious effects occur mainly due to the oxidation of proteins, DNA, and lipids, ultimately culminating in cell death [53,54]. 

A balance between ROS levels and the activity of inactivating (antioxidant) enzymes is crucial for the maintenance of cellular homeostasis. Erythroid-related nuclear factor 2 (Nrf2) is a transcription factor that plays an important role in the response to oxidative stress to maintain redox balance. Under homeostatic conditions, Nrf2 is bound to its chaperone Keap1 (Kelch-like ECH association protein 1) in the cytoplasm. However, when oxidative stress occurs, Nrf2 dissociates from the inactive Keap1-Nrf2 complex and translocates to the nucleus, where it regulates specific gene expression to induce the synthesis of antioxidant enzymes [55]. O_2_^−^ and H_2_O_2_ are inactivated by superoxide dismutase and catalase or the glutathione peroxidase system, respectively. OH**^.^** is typically more harmful than these ROS, as this oxygen-derived free radical does not have an intracellular inactivator. Its production intensifies the severity of injuries to cell structures, causing DNA damage caused by adducts of lipid peroxidation, and the production of other free radicals (such as malondialdehyde, hydroperoxide, and ONOO^−^, among other substances capable of stimulating the adherence of granulocytes to the microvascular endothelium [55,56]. 

### 2.3. Molecular Probe Fundamentals

Oxidative and nitrosative stress biomarkers are important tools to assess the balance between reactive species and antioxidants, contributing to the understanding of the pathophysiology of diseases [57]. Direct measurement of your cellular levels is a challenge, as direct and accurate measurement is complex, due to its short productive life and fast reactivity with other REDOX regulators [58]. Fluorescent probes for ROS selectively assess cellular levels of ROS in a very simple way, but it is important to consider their limitations. Fluorescent probes are able to monitor the behavior of a target biomolecule in live cells in real time [59].

The dihydrorhodamine 123 (DHR123) probe passively diffuses the cell membrane and concentrates in the intracellular space. In the presence of H_2_O_2_, hypochlorous acid (HOCl), or ONOO^−^, it is oxidized to rhodamine (R123) which exhibits green fluorescence. DHR123 is considered an intracellular probe for general detection of ROS; however, it has a lower stability than several other commercially available probes.

The CM-H_2_DCFDA (5-diacetate and 6-chloromethyl-2′,7′-dichlorodihydro-fluorescein) probe passively crosses the plasma membrane to enter the cell after which its acetate groups are cleaved by esterases to generate intracellular CM-H_2_DCF; the thiol-reactive chloromethyl group reacts with intracellular glutathione and other thiols, and subsequent oxidation renders a fluorescent intracellular adduct. This probe is used to detect intracellular ROS and can react with H_2_O_2_, OH, ONOO^−^ and other peroxide radicals. However, it is easily auto-oxidized resulting in a spontaneous increase in fluorescence, which must be corrected for at the time of the reading, discounting the value of a cell-free well containing the probe, as described by Hempel et al. Although this type of probe mainly detects H_2_O_2_, OH, and ONOO^−^, it is not specific for any oxidant because it responds to a wide range of oxidizing reactions; the CM-H_2_DCFDA probe is therefore considered a probe for general detection of ROS [60].

Fluorogenic complex probes containing boronate are used as a basis for detecting intracellular H_2_O_2_. Aromatic boronates react with H_2_O_2_, to generate a corresponding phenol, forming a highly fluorescent molecule in cells. Arylboronates also react with ONOO^−^, six times faster than with H_2_O_2_, verified by flow kinetics technique and high performance liquid chromatography (HPLC) analysis [61]. One of the characteristics is its photophysical properties, such as high photostability and suitable high fluorescence. In addition, the iminocoumarin by-products have excitation and emission wavelengths that are longer, whereas rapid cyclization would generate the highly fluorescent benzothiazolyl iminocoumarin [62].

Amplex Red reagent is a colorless, highly sensitive, non-fluorescent compound used as a stable probe to detect the generation of H_2_O_2_. It is oxidized by horseradish peroxidase (HRP) to a fluorescent product, resorufin. One of the main complicating factors is photochemical oxidation in the presence of biological reducers (glutathione) that induce the formation of free radicals (O_2_^−^ and H_2_O_2_), making the measurement of intracellular H_2_O_2_ a problem, even in the absence of HRP and H_2_O_2_. It is a highly sensitive method for detecting H_2_O_2_ and resorufin is stable for some time. However, it is impervious to cells and cannot be used to detect intracellular H_2_O_2_. Amplex Red is a very sensitive method for detecting ROS in organelles, as well as extracellular ROS, which is freely diffusible. The Amplex Red assay is also used to evaluate ROS formation in mitochondria [61,63].

CellRox represents another class of probes used for the general detection of ROS and comes in different models capable of emitting distinct fluorescence signals. In a reduced state, these cell-permeant dyes are non- or weakly fluorescent and become fluorescent upon oxidation by ROS. In general, CellRox can be oxidized by OH. and O_2_^−^, while CellRox orange is also capable of detecting H_2_O_2_, NO and ONOO-. These probes exhibit outstanding photostability compared to DCF [64] and it has also been shown that these probes can detect signals not detected by DCF [65]. Furthermore, depending on the model, can be used for in situ detection, allowing the assessment of real-time ROS dynamics in any given tissue [66].

The dihydroethidium (DHE) probe is capable of specifically detecting O_2_^−^ radicals in intracellular and extracellular environment [67]. In addition, it can also be used to detect O_2_^−^ in situ. The primary radical hydroethidine is derived from the loss of an aromatic amino hydrogen atom that, upon rearrangement, further reacts with another O_2_^−^ anion to form DHE. Acetylation of the aromatic amino groups in hydroethidine inhibited its reaction with O_2_^−^ [68]. MitoSox is the preferred probe for the specific analysis of mitochondrial O_2_^−^; this reagent selectively targets mitochondria where it is rapidly oxidized by O_2_^−^ (but not by other ROS or RNS) producing a red fluorescent signal, the oxidized product is highly fluorescent upon binding to nucleic acid [69].

DAF-FM (4-amino-5-methylamino-2′,7′-difluorofluorescein diacetate) is the leading molecular probe for the detection of NO. Like CM-H_2_DCFDA, DAF-FM diacetate also passively diffuses the plasma membrane and is cleaved by esterases to generate intracellular DAF-FM. Subsequent oxidation by NO yields a triazole product accompanied by increased fluorescent recovery [70]. DAF-FM is not a reversible balance sensor, which limits its ability to track rapid target substance (NO) fluctuations in real time. 

The aminophenyl fluorescein (APF) and hydroxyphenyl fluorescein (HPF) probes provide better selectivity and stability than CM-H_2_DCFDA for specific detection of OH^.^ and ONOO^−^ with relatively high resistance to light-induced oxidation. In their initial (reduced) form, the APF and HPF molecular probes are not fluorescent until they react with ONOO^−^ or OH, producing bright green fluorescence [45], resulting in cleavage of the aminophenyl ring from the fluorescein ring system, which is highly fluorescent. APF will also be transformed into the fluorescent form if exposed to a combination of H_2_O_2_ and horseradish peroxidase (HRP); HRP catalyzes the oxidation of APF by H_2_O_2_ [71].

The main fluorescent probes are widely used, mainly due to their simplicity, sensitivity, selectivity, execution speed and wide possibility of use in liquids and organic materials. There are limitations that should be known, such as autoxidation, but this could possibly be mitigated by the combined use of several fluorescent probes. Some regularly used probes are listed in Table 1.

### 2.4. Translational Studies

Molecular fluorescent probes for the detection of free radicals have been increasingly used in experimental animal studies and clinical trials, with proof of diagnostic efficacy in injuries resulting from visceral ischemia and reperfusion in a range of diseases. Childs and co-workers (2002) conducted a study with a fluorescent probe sensitive to hydroperoxides (DHR123) in Sprague-Dawley rats submitted to hemorrhagic shock. They evaluated the production of ROS in real-time and demonstrated an 80% elevation 5 min into the reperfusion phase, followed by an increase in leukocyte adherence between 5 and 10 min of reperfusion after volume replacement [73]. Others recently reported the attenuation of oxidative damage as measured by the fluorescent probe DCFH-DA (2′,7′-dichlorodihydrofluorescein diacetate) in an experimental model of reperfusion brain injury in rats [74]. In rats subjected to 45 min of the celiac trunk and superior mesenteric artery ischemia, followed by 60 min of reperfusion, treatment with melatonin (applied 5 min before to reperfusion) significantly reduced ischemia-reperfusion injury (neutrophil-mediated oxidative stress) as indicated by the inhibition of pathways related to ONOO^−^ measured by the molecular probe DHR123 [75]. Yan et al. used the DCFH-DA fluorescent probe to confirm the attenuation of oxidative stress induced by temporary ischemia of the superior mesenteric artery in mice treated with HO-1-expressing bone mesenchymal stromal cells (BMSC); based on the analysis using the fluorescent probe, it was concluded that BMSC that express HO-1 are more effective than treatment with BMSC alone in limiting intestinal damage and inflammation following ischemia and reperfusion injury [76]. Nagira et al. used DHR123 in the monolayers of human intestinal epithelial cell line to indicate that tight junctions and dysfunction of P-glycoprotein are induced through generation of reactive oxygen metabolites by ischemia and reperfusion in vitro model, and demonstrate the use of lutein as an antioxidant [77].

**Figure 2:**
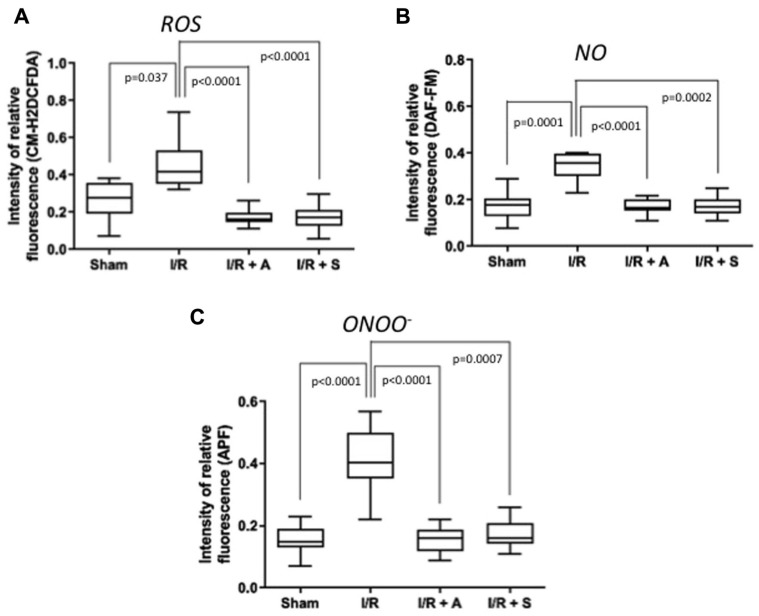
Sulforaphane (S) and albumin (A) administration attenuates the production of reactive oxygen and nitrogen species in intestinal ischemia/ reperfusion (I/R) injury. The administration of S and A before reperfusion prevented increases in reactive oxygen species (ROS) (**A**), nitric oxide (NO) (**B**), and peroxynitrite (ONOOL) (**C**) in the peripheral blood. The horizontal bars represent the medians, the boxes represent the 25th and 75th percentiles, and the vertical lines below and above the boxes represent the minimum and maximum values, respectively. The data are representative of two independent experiments (8 animals per group) [78]. The value of each “*p*” is showing in the figure its value related to the groups shown in blox plot graph.

Recently was performed a study with Wistar rats in a model of small bowel ischemia (established by clamping of branches of the superior mesenteric artery) followed by reperfusion. Using fluorescent molecular probes, we measured the synthesis of ROS and RNS, 80 min after starting the experiment and 45 min after reperfusion. The CM-H_2_DCFDA probe was used for general analysis of intracellular ROS, whereas DAF-FM and APF allowed specific evaluation of NO and ONOO^−^, respectively. Analysis of the results using these fluorescent probes revealed that treatment with the antioxidants sulforaphane and albumin significantly reduced levels of total ROS, NO, and ONOO^−^ in rats subjected to intestinal ischemia and reperfusion. Furthermore, reduced formation of free radicals and their by-products was shown to protect the intestinal mucosa. Antioxidant treatment decreased the concentration of macrophage-positive cells (ED-1), activation of intracellular NFκB signaling, and increased the amount of iNOS, LDH, and caspase 3 expression. They also observed relevant intestinal mucosal lesions and reduced concentration of goblet cells, a significant increase in apoptosis, greater macrophage infiltration, detachment, and structural disarrangement of the small intestine epithelium [78] (Figure 2).

The development of new versatile fluorescent probes with the possibility of high yield, high photostability, fast response time, low detection limit, high sensitivity and selectivity, low cytotoxicity, is what has been pursued by research aimed at diagnosing and interpreting evolution [79]. It is imperative for redox researchers to understand the detection mechanism and limitations of fluorescent probes in order to draw appropriate conclusions.

A clinically useful probe to identify biomarker(s) of mesenteric ischemia should have diagnostic specificity, exhibit prognostic value, be reasonably stable in various biological samples, and correlate with disease severity. Application and measurements would also need to be cost-effective with high reproducibility. Despite dozens of recognized markers and methods, results using fluorescent probes for the detection of oxidative stress are inconsistent among authors and thus weaken the overall translational value for clinical-surgical practice [80]. Therefore, additional and uniform research with consistent sampling will be necessary to avoid biases and identify the limited values of molecular probes as well as disease-specific diagnostic standards. Concerning AMI, future investigations using selective fluorescent probes, in parallel with proteomic and metabolomic approaches, will considerably improve our understanding of the signaling mechanisms that underpin the disease and facilitate the identification of clinically relevant biomarkers.

Table 2 lists some studies that support the use of fluorescent probes, especially in the pathophysiology of ischemia reperfusion, based on various clinical conditions, showing benefits from their use. What we need is the translational extrapolation to clinical practice, with clinical works that support the proper use in some specific conditions, mainly because there is a technological effort to improve the quality of fluorescent probes. Acting on the pathophysiological basis of some diseases seems to be better supported.

## Figures and Tables

**Table 1:** Probes for reactive species [72]. In the table are represented some fluorescent probes and possible reactive species identified in each reaction.

Probes	Reactive Species	Chemical Structure
DHR123	Hydrogen peroxide Hypochlorous acid Peroxynitrite anion	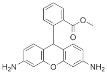
CM-H_2_DCFDA	Hydrogen peroxide Hydroxyl radical Peroxynitrite anion Peroxyl radical	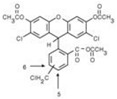
CellRox	Hydrogen peroxide Hydroxyl radical Nitric oxide Peroxynitrite anion Superoxide anion	
Dihydroethidium	Superoxide anion	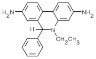
MitoSox	Superoxide anion	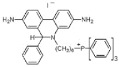
DAF-FM	Nitric oxide	
APF and HPF	Hypochlorous acid Peroxynitrite anion	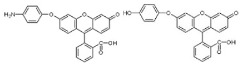
	Hydroxyl radical	
Boronate	Hydrogen peroxide Peroxynitrite anion	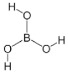
Amplex red	Hydrogen peroxide	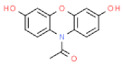

**Table 2:** Fluorescent probes for reactive oxygen species use in translational studies. Several studies have been carried out to justify the use of fluorescent probes in experimental models with the possibility of use in clinical practice.

Author	Fluorescent Probe	Results	Graphics
Childs EW, et al. [73]	Dihydrorhodamine 123 i.v. and observes in vivo mesenteric endothelium	Reactive oxygen species production in the mesenteric microvascular endothelium, attributed to hemorrhagic shock and reperfusion injury, after resuscitation, and mediated by the administration of a platelet activating factor antagonist	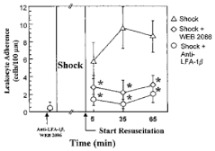 Effect of platelet activating factor (anti-LFA-1_ and WEB 2086) on leukocyte adherence ROS given 10 min prior to the shock period versus the hemorrhagic shock alone group. * *p* < 0.05 compared with the hemmorhagic shock alone group.
Tang Y, et al. [74]	DCFH-DA (2′, 7′-dichlorodihydrofluorescein diacetate) used in fresh tissue homogenates	Human albumin intravenous administration, in ROS attenuation, in a global cerebral ischemia reperfusion model by Wnt/β-Catenin pathway signaling	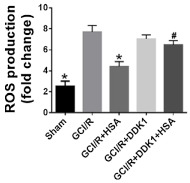 Effect of human albumin treatment on oxidative stress following global cerebral ischemia/reperfusion, (*p* < 0.05) in contrast to the Global Cerebral Ischemia/Reperfusion (GCI/R) group, * *p* < 0.05 in contrast to GCI/R group, # *p* < 0.05, in contrast to the GCI/R+Human Serum Albumin group.
Cuzzocrea S, et al. [75]	Dihydrorodamine 123 i.v. plasma analysed	Melatonin infusion attenuated the reperfusion injury produced by splanchnic artery occlusion	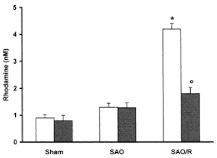 Plasma peroxynitrite production assessed by oxidation of dihydrorhodamine 123 to rhodamine. Peroxynitrite production in the Splancnic Arterial Oclusion (SAO)-shocked rats was significantly increased versus sham group. Melatonin-treated rats show a significant reduction of the SAO-induced elevation of the plasma peroxynitrite production. * *p* < 0.01 versus vehicle. ° *p* < 0.01 versus SAO.
Yan XT, et al. [76]	DCFH-DA used in homogenized intestinal tissue	Heme Oxygenase-1-expressing Bone Marrow Steam Cell after intestinal I/R performed by temporary occlusion of the superior mesenteric artery	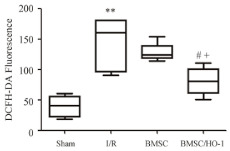 Bone Marrow Steam Cell/HemeOxygenase-1 (BMSC/HO-1) attenuated production of ROS in intestine and serum. Levels of ROS in intestine at 24 h of reperfusion were significantly higher than those in sham group and decreased after treatment of BMSC/HO-1. ** *p* < 0.01 vs. Sham; # *p* < 0.05 vs. I/R; + *p* < 0.05 vs. BMSC.
Nagira M, et al. [77]	Rhodamine 123	Lutein effects In vitro ischemia reperfusion injury, using monolayers of human colon cancer intestinal epithelial cell line	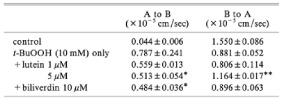 The effects of lutein and biliverdin on rhodamine 123 permeability in the apical to basal direction in cell monolayers. * *p* < 0.05 and ** *p* < 0.01 vs. lipid peroxidation inducer.

## References

[1] Kuhn F., Schiergens T.S., Klar E. (2020). Acute mesenteric ischemia. Visc. Med..

[2] Ehlert B.A. (2018). Acute gut ischemia. Surg. Clin. North Am..

[3] Karkkainen J.M. (2021). Acute mesenteric ischemia: A challenge for the acute care surgeon. Scand. J. Surg..

[4] Gnanapandithan K., Feuerstadt P. (2020). Review article: Mesenteric ischemia. Curr. Gastroenterol. Rep..

[5] Memet O., Zhang L., Shen J. (2019). Serological biomarkers for acute mesenteric ischemia. Ann. Transl. Med..

[6] Abdal Dayem A., Hossain M.K., Lee S.B., Kim K., Saha S.K., Yang G.M., Choi H.Y., Cho S.-G. (2017). The role of reactive oxygen species (ROS) in the biological activities of metallic nanoparticles. Int. J. Mol. Sci..

[7] Dhatt H.S., Behr S.C., Miracle A., Wang Z.J., Yeh B.M. (2015). Radiological evaluation of bowel ischemia. Radiol. Clin. N. Am..

[8] Peoc’h K., Corcos O. (2019). Biomarkers for acute mesenteric ischemia diagnosis: State of the art and perspectives. Ann. Biol. Clin..

[9] Haber F., Willstätter R. (1931). Unpaarigkeit und Radikalketten im Reaktionsmechanismus organischer und enzymatischer Vorgänge. Ber. Der Dtsch. Chem. Ges. A B Ser..

[10] Haber F., Weiss J., Pope W.J. (1934). The catalytic decomposition of hydrogen peroxide by iron salts. Proc. R. Soc. Lond. Ser. A Math. Phys. Sci..

[11] McCord J.M., Fridovich I. (1969). Superoxide dismutase. An enzymic function for erythrocuprein (hemocuprein). J. Biol. Chem..

[12] De Flora S., Grassi C., Carati L. (1997). Attenuation of influenza-like symptomatology and improvement of cell-mediated immunity with long-term *N*-acetylcysteine treatment. Eur. Respir. J..

[13] Acosta S. (2015). Mesenteric ischemia. Curr. Opin. Crit. Care..

[14] Karkkainen J.M., Acosta S. (2017). Acute mesenteric ischemia (part I)—Incidence, etiologies, and how to improve early diagnosis. Best Pract. Res. Clin. Gastroenterol..

[15] Karkkainen J.M., Acosta S. (2017). Acute mesenteric ischemia (Part II)—Vascular and endovascular surgical approaches. Best Pract. Res. Clin. Gastroenterol..

[16] Prakash V.S., Marin M., Faries P.L. (2019). Acute and chronic ischemic disorders of the small bowel. Curr. Gastroenterol. Rep..

[17] Granger D.N. (1981). Intestinal microcirculation and transmucosal fluid transport. Am. J. Physiol..

[18] Granger D.N., Hollwarth M.E., Parks D.A. (1986). Ischemia-reperfusion injury: Role of oxygen-derived free radicals. Acta Physiol. Scand. Suppl..

[19] Kajino-Sakamoto R., Omori E., Nighot P.K., Blikslager A.T., Matsumoto K., Ninomiya-Tsuji J. (2010). TGF-beta-activated kinase 1 signaling maintains intestinal integrity by preventing accumulation of reactive oxygen species in the intestinal epithelium. J. Immunol..

[20] VanDeinse W.H., Zawacki J.K., Phillips D. (1986). Treatment of acute mesenteric ischemia by percutaneous transluminal angioplasty. Gastroenterology.

[21] Beaulieu R.J., Arnaoutakis K.D., Abularrage C.J., Efron D.T., Schneider E., Black J.H. (2014). Comparison of open and endovascular treatment of acute mesenteric ischemia. J. Vasc. Surg..

[22] Yan L., Xie Y., Li J. (2019). A colorimetric and fluorescent probe based on rhodamine B for detection of Fe(3+) and Cu(2+) ions. J. Fluoresc..

[23] Nadatani Y., Watanabe T., Shimada S., Otani K., Tanigawa T., Fujiwara Y. (2018). Microbiome and intestinal ischemia/reperfusion injury. J. Clin. Biochem. Nutr..

[24] Bertoni S., Ballabeni V., Barocelli E., Tognolini M. (2018). Mesenteric ischemia-reperfusion: An overview of preclinical drug strategies. Drug Discov. Today.

[25] Mester A., Magyar Z., Sogor V., Tanczos B., Stark Y., Cherniavsky K., Laszlo B., Katalin P., Norbert N. (2018). Intestinal ischemia-reperfusion leads to early systemic micro-rheological and multiorgan microcirculatory alterations in the rat. Clin. Hemorheol. Microcirc..

[26] Papezikova I., Lojek A., Cizova H., Ciz M. (2006). Alterations in plasma antioxidants during reperfusion of the ischemic small intestine in rats. Res. Vet. Sci..

[27] Kalogeris T., Baines C.P., Krenz M., Korthuis R.J. (2016). Ischemia/reperfusion. Compr. Physiol..

[28] Granger D.N., Kvietys P.R. (2015). Reperfusion injury and reactive oxygen species: The evolution of a concept. Redox. Biol..

[29] Wu Z., Wang H., Fang S., Xu C. (2018). Roles of endoplasmic reticulum stress and autophagy on H_2_O_2_ induced oxidative stress injury in HepG2 cells. Mol. Med. Rep..

[30] Valko M., Morris H., Cronin M.T. (2005). Metals, toxicity and oxidative stress. Curr. Med. Chem..

[31] Liskova A., Samec M., Koklesova L., Kudela E., Kubatka P., Golubnitschaja O. (2021). Mitochondriopathies as a clue to systemic disorders-analytical tools and mitigating measures in context of predictive, preventive, and personalized (3P) medicine. Int. J. Mol. Sci..

[32] Battelli M.G., Polito L., Bolognesi A. (2014). Xanthine oxidoreductase in atherosclerosis pathogenesis: Not only oxidative stress. Atherosclerosis.

[33] Kleinert H., Schwarz P.M., Forstermann U. (2003). Regulation of the expression of inducible nitric oxide synthase. Biol. Chem..

[34] Valenca S.S., Pimenta W.A., Rueff-Barroso C.R., Ferreira T.S., Resende A.C., Moura R.S., Porto L.C. (2009). Involvement of nitric oxide in acute lung inflammation induced by cigarette smoke in the mouse. Nitric Oxide.

[35] Pires K.M., Lanzetti M., Rueff-Barroso C.R., Castro P., Abrahao A., Koatz V.L., Valença S.S., Porto L.C. (2012). Oxidative damage in alveolar macrophages exposed to cigarette smoke extract and participation of nitric oxide in redox balance. Toxicol. In Vitro.

[36] Nesi R.T., Barroso M.V., Souza Muniz V., de Arantes A.C., Martins M.A., Brito Gitirana L., Neves J.S., Benjamim C.F., Lanzetti M., Valenca S.S. (2017). Pharmacological modulation of reactive oxygen species (ROS) improves the airway hyperresponsiveness by shifting the Th1 response in allergic inflammation induced by ovalbumin. Free Radic Res..

[37] Valenca S.S., Rueff-Barroso C.R., Pimenta W.A., Melo A.C., Nesi R.T., Silva M.A., Porto L.C. (2011). L-NAME and L-arginine differentially ameliorate cigarette smoke-induced emphysema in mice. Pulm. Pharmacol. Ther..

[38] Lanzetti M., da Costa C.A., Nesi R.T., Barroso M.V., Martins V., Victoni T., Lagente V., Pires K.M.P., Silva P.M.R.e., Resende A.C. (2012). Oxidative stress and nitrosative stress are involved in different stages of proteolytic pulmonary emphysema. Free Radic. Biol. Med..

[39] Li H., Forstermann U. (2000). Nitric oxide in the pathogenesis of vascular disease. J. Pathol..

[40] Guzik T.J., Korbut R., Adamek-Guzik T. (2003). Nitric oxide and superoxide in inflammation and immune regulation. J. Physiol. Pharmacol..

[41] Moller M.N., Rios N., Trujillo M., Radi R., Denicola A., Alvarez B. (2019). Detection and quantification of nitric oxide-derived oxidants in biological systems. J. Biol. Chem..

[42] Barzilai A., Yamamoto K. (2004). DNA damage responses to oxidative stress. DNA Repair..

[43] Luo C.C., Huang C.S., Ming Y.C., Chu S.M., Chao H.C. (2016). Calcitonin gene-related peptide downregulates expression of inducible nitride oxide synthase and caspase-3 after intestinal ischemia-reperfusion injury in rats. Pediatr. Neonatol..

[44] Eiserich J.P., Patel R.P., O’Donnell V.B. (1998). Pathophysiology of nitric oxide and related species: Free radical reactions and modification of biomolecules. Mol. Aspects Med..

[45] Adams L., Franco M.C., Estevez A.G. (2015). Reactive nitrogen species in cellular signaling. Exp. Biol. Med..

[46] Harris C.M., Sanders S.A., Massey V. (1999). Role of the flavin midpoint potential and NAD binding in determining NAD versus oxygen reactivity of xanthine oxidoreductase. J. Biol. Chem..

[47] Laursen J.B., Somers M., Kurz S., McCann L., Warnholtz A., Freeman B.A., Tarpey M., Fukai T., Harrison D.G. (2001). Endothelial regulation of vasomotion in apoE-deficient mice: Implications for interactions between peroxynitrite and tetrahydrobiopterin. Circulation.

[48] Stocker R., Perrella M.A. (2006). Heme oxygenase-1: A novel drug target for atherosclerotic diseases?. Circulation.

[49] Huang Y., Wu Z., Riwanto M., Gao S., Levison B.S., Gu X., Fu X., Wagner M.A., Besler C., Gerstenecker G. (2013). Myeloperoxidase, paraoxonase-1, and HDL form a functional ternary complex. J. Clin. Investig..

[50] Schiffrin E.L. (2004). Remodeling of resistance arteries in essential hypertension and effects of antihypertensive treatment. Am. J. Hypertens..

[51] Nauseef W.M. (2004). Assembly of the phagocyte NADPH oxidase. Histochem. Cell Biol..

[52] Lee M.Y., Griendling K.K. (2008). Redox signaling, vascular function, and hypertension. Antioxid. Redox Signal..

[53] Liao G., Chen S., Cao H., Wang W., Gao Q. (2019). Review: Acute superior mesenteric artery embolism: A vascular emergency cannot be ignored by physicians. Medicine.

[54] Singh M., Long B., Koyfman A. (2017). Mesenteric ischemia: A deadly miss. Emerg. Med. Clin. North Am..

[55] Li R., Jia Z., Zhu H. (2019). Regulation of Nrf2 signaling. React. Oxyg. Species.

[56] Kehrer J.P. (2000). The Haber-Weiss reaction and mechanisms of toxicity. Toxicology.

[57] Duanghathaipornsuk S., Farrell E.J., Alba-Rubio A.C., Zelenay P., Kim D.S. (2021). Detection technologies for reactive oxygen species: Fluorescence and electrochemical methods and their applications. Biosensors.

[58] Katerji M., Filippova M., Duerksen-Hughes P. (2019). Approaches and methods to measure oxidative stress in clinical samples: Research applications in the cancer field. Oxid. Med. Cell Longev..

[59] Jiang X., Wang L., Carroll S.L., Chen J., Wang M.C., Wang J. (2018). Challenges and opportunities for small-molecule fluorescent probes in redox biology applications. Antioxid. Redox Signal..

[60] Hempel S.L., Buettner G.R., O’Malley Y.Q., Wessels D.A., Flaherty D.M. (1999). Dihydrofluorescein diacetate is superior for detecting intracellular oxidants: Comparison with 2′,7′-dichlorodihydrofluorescein diacetate, 5(and 6)-carboxy-2′,7′-dichlorodihydrofluorescein diacetate, and dihydrorhodamine 123. Free Radic. Biol. Med..

[61] Kalyanaraman B., Darley-Usmar V., Davies K.J., Dennery P.A., Forman H.J., Grisham M.B., Mann G.E., Moore K., Roberts J., Ischiropoulss H. (2012). Measuring reactive oxygen and nitrogen species with fluorescent probes: Challenges and limitations. Free Radic. Biol. Med..

[62] Li M., Han H., Zhang H., Song S., Shuang S., Dong C. (2020). Boronate based sensitive fluorescent probe for the detection of endogenous peroxynitrite in living cells. Spectrochim. Acta A Mol. Biomol. Spectrosc..

[63] Deshwal S., Antonucci S., Kaludercic N., di Lisa F. (2018). Measurement of mitochondrial rOS Formation. Methods Mol. Biol..

[64] (2011). Fluorescence imaging of oxidative stress in live cells. BioProbes J. Cell Biol. Appl..

[65] Schenk B., Fulda S. (2015). Reactive oxygen species regulate Smac mimetic/TNFalpha-induced necroptotic signaling and cell death. Oncogene.

[66] Kageyama S., Hirao H., Nakamura K., Ke B., Zhang M., Ito T., Aziz A., Oncel D., Kaldas F.M., Bussutil R.W. (2019). Recipient HO-1 inducibility is essential for posttransplant hepatic HO-1 expression and graft protection: From bench-to-bedside. Am. J. Transplant..

[67] Peshavariya H.M., Dusting G.J., Selemidis S. (2007). Analysis of dihydroethidium fluorescence for the detection of intracellular and extracellular superoxide produced by NADPH oxidase. Free Radic. Res..

[68] Zhao H., Joseph J., Fales H.M., Sokoloski E.A., Levine R.L., Vasquez-Vivar J., Kalyanaraman B. (2005). Detection and characterization of the product of hydroethidine and intracellular superoxide by HPLC and limitations of fluorescence. Proc. Natl. Acad. Sci. USA.

[69] Dikalov S.I., Harrison D.G. (2014). Methods for detection of mitochondrial and cellular reactive oxygen species. Antioxid. Redox Signal.

[70] Nagano T. (2009). Bioimaging probes for reactive oxygen species and reactive nitrogen species. J. Clin. Biochem. Nutr..

[71] Cohn C.A., Simon S.R., Schoonen M.A. (2008). Comparison of fluorescence-based techniques for the quantification of particle-induced hydroxyl radicals. Part Fibre Toxicol..

[72] Wiederschain G.Y. (2011). The molecular probes handbook. A guide to fluorescent probes and labeling technologies. Biochemistry.

[73] Childs E.W., Udobi K.F., Wood J.G., Hunter F.A., Smalley D.M., Cheung L.Y. (2002). In vivo visualization of reactive oxidants and leukocyte-endothelial adherence following hemorrhagic shock. Shock.

[74] Tang Y., Shen J., Zhang F., Yang F.Y., Liu M. (2019). Human serum albumin attenuates global cerebral ischemia/reperfusion-induced brain injury in a Wnt/beta-Catenin/ROS signaling-dependent manner in rats. Biomed. Pharmacother..

[75] Cuzzocrea S., Costantino G., Mazzon E., Micali A., de Sarro A., Caputi A.P. (2000). Beneficial effects of melatonin in a rat model of splanchnic artery occlusion and reperfusion. J. Pineal Res..

[76] Yan X.T., Cheng X.L., He X.H., Zheng W.Z., Xiao-Fang Y., Hu C. (2019). The HO-1-expressing bone mesenchymal stem cells protects intestine from ischemia and reperfusion injury. BMC Gastroenterol..

[77] Nagira M., Tomita M., Mizuno S., Kumata M., Ayabe T., Hayashi M. (2006). Ischemia/reperfusion injury in the monolayers of human intestinal epithelial cell line caco-2 and its recovery by antioxidants. Drug Metab. Pharmacokinet..

[78] Sampaio de Holanda G., dos Santos Valenca S., Maran Carra A., Lopes Lichtenberger R.C., Franco O.B., Ribeiro B.E., Rosas S.L.P., Santana P.T., Castelo-Branco M.T.L., de Souza H.S.F. (2021). Sulforaphane and albumin attenuate experimental intestinal ischemia-reperfusion injury. J. Surg. Res..

[79] Fang Y., Dehaen W. (2021). Fluorescent probes for selective recognition of hypobromous acid: Achievements and future perspectives. Molecules.

[80] Frijhoff J., Winyard P.G., Zarkovic N., Davies S.S., Stocker R., Cheng D., Knight A.R., Taylor E.L., Oettrich J., Ruskovska T. (2015). Clinical relevance of biomarkers of oxidative stress. Antioxid. Redox Signal.

